# IL-3 produced by T cells is crucial for basophil extravasation in hapten-induced allergic contact dermatitis

**DOI:** 10.3389/fimmu.2023.1151468

**Published:** 2023-04-26

**Authors:** Carole El Hachem, Pierre Marschall, Pierre Hener, Anupama Karnam, Srinivasa Reddy Bonam, Pierre Meyer, Eric Flatter, Marie-Christine Birling, Jagadeesh Bayry, Mei Li

**Affiliations:** ^1^ Institut de Génétique et de Biologie Moléculaire et Cellulaire, Centre National de la Recherche Scientifique UMR7104, Institut National de la Santé et de la Recherche Médicale U1258, Université de Strasbourg, Illkirch, France; ^2^ Institut National de la Santé et de la Recherche Médicale, Centre de Recherche des Cordeliers, Sorbonne Université, Université de Paris, Paris, France; ^3^ Institut Clinique de la Souris, Illkirch, France; ^4^ Department of Biological Sciences & Engineering, Indian Institute of Technology Palakkad, Palakkad, India

**Keywords:** basophil, IL-3, allergy, skin, extravasation, integrin, retinoic acid

## Abstract

Basophils have been recognized as a characterized cellular player for Th2 immune responses implicated in allergic diseases, but the mechanisms responsible for basophil recruitment to allergic skin remain not well understood. Using a hapten fluorescein isothiocyanate (FITC)-induced allergic contact dermatitis (ACD) mouse model, we show that basophils in FITC-treated IL-3-knockout mice are defective in crossing the vascular endothelium to enter the inflamed skin. By generating mice in which IL-3 is selectively ablated in T cells, we further demonstrate that IL-3 produced by T cells mediates basophil extravasation. Moreover, basophils sorted from FITC-treated IL-3-knockout mice exhibit a decreased expression of integrins Itgam, Itgb2, Itga2b and Itgb7, which are potentially implicated in extravasation process. Interestingly, we observed that these basophils had a reduced expression of retinaldehyde dehydrogenase 1 family member A2 (Aldh1a2), an enzyme responsible for the production of retinoic acid (RA), and administration of all-trans RA restored partially the extravasation of basophils in IL-3-knockout mice. Finally, we validate that IL-3 induces the expression of ALDH1A2 in primary human basophils, and provide further evidence that IL-3 stimulation induces the expression of integrins particularly ITGB7 in an RA-dependent manner. Together, our data propose a model that IL-3 produced by T cells activates ALDH1A2 expression by basophils, leading to the production of RA, which subsequently induces the expression of integrins crucially implicated in basophil extravasation to inflamed ACD skin.

## Introduction

Basophils, one type of circulating granulocytes that account less than 1% of peripheral blood leukocytes, represent a characteristic cellular component in parasite infection and allergic skin inflammation. Basophils complete their maturation in the bone marrow, circulate in the blood and migrate to tissue under inflammatory conditions. They have been shown to infiltrate skin lesions in certain skin disorders such as allergic contact dermatitis (ACD), acute atopic dermatitis (AD), prurigo, urticaria and bullous pemphigoid, but are absent in other skin disorders like psoriasis vulgaris (1).

Despite of being the least abundant circulating leukocytes, basophils have been recognized to play important roles in physiological and pathological contexts. Basophils are recruited to inflamed tissues and activated in an IgE-dependent or -independent manner to release a variety of effector molecules, such as histamine and leukotriene C4, chemotactic factors, and cytokines including IL-4, IL-13 that are involved in immediate and late-phase reactions of the immune system (2). In addition, basophils were reported to crosstalk with other inflammatory cells, for example to mediate eosinophil recruitment to allergic skin (3, 4) or to confer an M2-like phenotype on macrophages (5).

Although our knowledge on basophil function has been rapidly expanded, how these cells infiltrate to inflammatory sites remains not well understood. IL-3 has been implicated in basophil survival *in vitro* (6), and activation (7, 8), in regulating basophil expansion in blood, or basophil production from the bone marrow in *Nippostrongylus brasilensis (N.b.)* parasite infection mouse models (9, 10). IL-3 was also reported to play a role for basophil recruitment to the mesenteric lymph nodes in *N.b.* infection (Kim et al., 2010), or to skin-draining lymph nodes in an AD mouse model (11). Yet, it remained not defined how important IL-3 is for basophil recruitment to allergic skin site and what are underlying mechanisms.

Tissue inflammatory immune response develops upon the extravasation of leukocytes into the tissue by crossing blood vessels. For circulating leukocytes to enter a tissue under inflammatory conditions, a cascade of events is required that involves an interaction between the leukocyte and endothelial cells (ECs), comprising essential sequential steps including chemo-attraction, rolling, adhesion to the blood vessel wall and trans-endothelial migration (TEM): first, triggering of the activation of leukocyte rolling and adhesion by chemokines (12); second, the binding of selectins (P-and E-selectins on the endothelium) to their ligands such as P-selectin glycoprotein ligand 1 (PSGL-1) expressed by leukocytes, and regulation of leukocyte rolling on the endothelium; third, adhesion of leukocytes to blood vessels by intergrins expressed on leukocyte surface to bind to their ligands expressed on ECs (e.g. ICAM-1, VCAM-1…); finally, TEM where leukocytes cross ECs lining the blood vessels (13, 14).

Integrins have been identified as important molecules implicated in leukocyte extravasation. Integrins are composed of a complex family of αβ heterodimers that can assemble into different receptors in vertebrates (15). For example, ITGAL/ITGB2 and ITGAM/ITGB2 were shown to be involved in neutrophil extravasation (16, 17) and ITGA4/ITGB7 for T cell migration (18). As to basophil extravasation, *in vitro* studies have shown that IL-3 receptor complex is expressed in ECs or basophils (19, 20), and treatment of ECs (21) or basophils (22, 23) with IL-3 enhanced basophil rolling, adhesion and TEM. Antibodies against PSGL-1, P-selectin, ITGAM, ITGB2 or ITGB1 were shown to inhibit basophil adhesion and migration to ECs (21–23). However, all these studies were performed *in vitro* and there was little *in vivo* study to explore basophil extravasation to inflamed tissues.

In this study, we investigated basophil recruitment in allergic skin by using hapten FITC-induced ACD mouse model (24), where basophil infiltration is a characterized feature. We demonstrate a crucial role of IL-3 produced by T cells in mediating basophil extravasation to the inflamed skin, and show that in the absence of IL-3 signaling, basophils exhibit reduced expression of a number of integrins that was accompanied by a reduced expression of retinoic acid (RA)-producing enzyme ALDH1A2. We tested whether the supplement of RA restores basophil skin extravasation in IL-3-knockout mice, and further examined the potential role of RA signaling in the regulation of integrins in IL-3-stimulated human primary basophils. Our data thus provide insights on a central role of IL-3 in the interaction between T cells, basophils and ECs in mediating basophil extravasation to the inflamed skin.

## Materials and methods

### Mice

Wild-type BALB/c mice were purchased from Charles River Laboratories. CD4-Cre^Tg/0^ mice (25) were purchased from the Jackson laboratory and were backcrossed into Balb/c background (>99%).

IL-3-ablated (*Il3^-/-^*) mice and -floxed (*Il3^L2/L2^*) mice (all in pure Balb/c background) were generated by us at the Institut Clinique de la Souris (ICS) (**Figure S1**). In order to obtain an Il3 “2 in 1” allele (tm1a, **Figure S1**), we acquired and modified an IMPC plasmid ETPG00275_W_2_F02 (https://www.mousephenotype.org/data/genes/MGI:96552). This plasmid was digested with a RsrII restriction enzyme to remove the LacZ and the 5’ region of the NeoR cassette, and a DNA fragment containing the eGFP cDNA and the deleted part (5’ region) of the NeoR cassette (ordered from GeneArt) was amplified with primers containing 25 bps homology for the IMPC vector and cloned to the plasmid using the SLIC method (26). The resulting plasmid was fully sequenced to confirm the presence of all the desired components including *in frame* eGFP, Lox and FRT sites and NeoR cassette. After cutting with PvuI, the linearized construct was electroporated in in-house derived BALB/CN mouse embryonic stem cells (ESCs). After selection, targeted clones were identified by PCR using external primers and were further confirmed by Southern blot using both a Neo probe (5’ and 3’ digests) as well as a 3’ external probe. Two positive ES clones were microinjected into C57BL/6N blastocysts. Resulting male chimeras were bred with wildtype C57BL/6N females. Germline transmission of the tm1a allele was obtained. The tm1c allele (or “L2” allele) was obtained after breeding of the heterozygous animal with a PHENOMIN-ICS BALC/CN Flp delete mouse line (**Figure S1**). The tm1b allele (GFP-KI/Il3-KO, or mutant “-” allele) was obtained after breeding the heterozygous animals with a PHENOMIN-ICS Cre deleter mouse line (**Figure S1**).

Breeding and maintenance of mice were performed under institutional guidelines, and all of the experimental protocols were approved by the animal care and ethics committee of animal experimentation of the IGBMC n°017 and by the Ministère de l’enseignement supérieur, de la recherche et de l’innovation.

### FITC treatment

Fluorescein isothiocyanate (FITC, ≥97.5% (HPLC) (Sigma) was first dissolved in acetone (to a concentration of 2%), then mixed with equal volume of dibutyl phthalate (DBP, Sigma) to get a final concentration of 1% FITC (in 1:1 DBP/acetone). Mice were sensitized with 25 μl of FITC (in 1:1 DBP/acetone) on the left ear (LE) followed by the challenge on the right ear (RE) with 25 μl of FITC (in 1:1 DBP/acetone), as indicated in experimental schemes in figures. RE thickness was measured using Digimatic Caliper (Mitutoyo).

### All-trans RA treatment

All trans-RA (at-RA; MP Biomedicals) was dissolved in ethanol for a stock solution (5 mg/ml; 16 mM). For topical treatment, at-RA was diluted in ethanol to a final concentration of 40 μM and topically applied on mouse ears (25 μl per ear); for intraperitoneal (i.p.) injection, 0.1 ml of RA (5 mg/ml in ETOH) was mixed with 4.9 ml of sunflower oil; vortexed and sonicated to make a solution with final concentration of 0.1 mg/ml for injection (10 μl/g mouse) (27).

### Cell preparation for FACS analyses

For preparation of dermal cells, ears were split into two halves, floated on a solution of Dispase (4mg/ml in PBS, Gibco) with epidermis side up, and incubated at 37°C for 1 h. Dermis was then separated from epidermis and was further incubated on an agitator at 37°C for 1 h in a solution containing 1 mg/ml collagenase D (Roche), 0.25 mg/mL DNaseI (Sigma) and 2.5% of foetal calf serum (FCS) (ThermoFisher) in PBS, then passed through a cell strainer (EASYstrainer 70 μm, Greiner bio-one). Cells were then centrifuged at 1200 rpm, 4°C for 5 min, resuspended in FACS buffer (1% of FCS + 2 mM EDTA in PBS), counted and used for FACS staining (2x10^6^ cells) or for sorting.

For preparation of blood cells, 400 μl of blood was collected from mice by retro-orbital bleeding in EDTA-coated tubes, mixed with the same volume of Dextran (2% in PBS, Sigma-Aldrich) and incubated for 30 min at 37°C. The upper phase was transferred into new tubes, 600 μl of FACS buffer was added, then centrifuged at 4000 rpm for 4 min at 4°C. The pellet was resuspended in 0.3 ml of ACK lysis buffer (Ammonium-Chloride-Potassium: NH4Cl 0.15 M; KHC03 1 mM; Na_2_EDTA 0.1 mM), incubated for 2 min at room temperature (RT), and then added 1ml of FACS buffer and centrifuged 4000 rpm for 4 min at 4°C. The pellet was resuspended in FACS buffer and used for FACS staining.

### Antibody staining and FACS analyses

Cells were first incubated with anti-mouse CD16/CD23 (Fc block) for 10 min on ice, then washed and stained with the surface antibodies (Abs, listed below), starting with biotinylated Abs in 25 μl of FACS buffer for 10 min on ice, then washed and stained with streptavidin mixed with other surface Abs in 25 μl of FACS buffer for 10 min on ice (except for CD34 Ab which was incubated for 90 min on ice). Cells were then washed with FACS buffer, incubated for 3 min with DAPI (final concentration: 1 μg/ml) for exclusion of dead cells before passing on LSRII (BD).

For intracellular staining, dermal cells were cultured in RMPI medium w/o HEPES, + 10% FCS +1% P/S and 2 mM Glutamin, in presence or absence of GolgiSTOP (BD) and Cell Stimulation Cocktail (eBioscience) at 37°C for 2 h. Cells were then washed with FACS buffer then incubated with anti-mouse CD16/CD23 (Fc block) for 10 min on ice, then washed with FACS buffer and stained with the surface Abs (listed below) as described above. Cells were then washed and resuspended with 100 μl of Fixation/Permeabilization solution (BD Cytofix/Cytoperm kit) for 20 min on ice, then washed twice with the wash buffer (BD Cytofix/Cytoperm kit). IL-3 Ab (listed below) was added and incubated on ice for 30 min. After washing, cells were finally resuspended with FACS buffer and passed on LSRII analyser.

Antibodies used for Flow cytometry are described in **Table 1**.

**Table 1:** Antibodies used for Flow cytometry.

Name	Fluorophore	Clone	Company	Dilution
CD16/CD32 (Fc block)		93	eBioscience	0.5:25
CD49b-biotin		DX5	eBioscience	0.5:25
IgE-biotin		R35-72	BD Biosciences	0.5:25
Streptavidin	BV605		Invitrogen	0.5:25
CD45	APC-eFluo780	30-F11	eBioscience	0.05:25
TCR-beta	PerCP-Cy5.5	H57-597	eBioscience	1:25
Siglec-F	Alexa Fluor647	E50-2440	BD Biosciences	1:25
Gr1	PE	RB6-8C5	eBioscience	0.02:25
CD34	eFluor 700	RAM34	eBioscience	4:25
ESAM-1	APC	1G8/ESAM	Biolegend	1.25:25
CD19	PerCP-Cy5.5	eBio1D3	eBioscience	1:25
CD3	FITC	145-2C11	eBioscience	1:25
CD45R/B220	PE-Cy7	RA3-6B2	Biolegend	1:25
IL-3	PE	MP2-8F8	Biolegend	1.25:50
FcϵRIα	Alexa Fluor 647	Fc23cpg	eBioscience	1:25

### RNA extraction of cells sorted from ears and quantitative RT-PCR

Ear dermal cells were prepared as described above. After antibody staining, cells were FACS-sorted: Endothelial cells (CD45^-^CD34^+^ESAM-1^+^), Hematopoietic cells (CD45^+^), TCRβ cells (CD45^+^TCRβ^+^), Neutrophils (CD45^+^TCRβ^-^Gr1^hi^), Eosinophils (CD45^+^TCRβ^-^Siglec-F^+^SSC^hi^), Basophils (TCRβ^-^Siglec-F^-^Gr1^-^CD45^lo^CD49b^+^). RNA was extracted with NucleoSpin RNA XS kit following the manufacturer’s instruction.

RNA was reverse transcribed by using random oligonucleotide hexamers and amplified by means of quantitative PCR with LightCycler 480 (Roche Diagnostics) and QuantiTect SYBR Green kit (Qiagen), according to the manufacturer’s instructions. Relative RNA levels were calculated with hypoxanthine phosphoribosyl- transferase (HPRT) as an internal control. Sequences of PCR primers for mouse genes are described in **Table 2**.

**Table 2:** Sequences of PCR primers for mouse genes.

Gene name	Sequence 5’ to 3’
*Hprt*	TGGATACAGGCCAGACTTTG GATTCAACTTGCGCTCATCTTA
*Il3*	TGAAGGACCCTCTCTGAGGA CGCAGATCATTCGCAGAT
*Il4*	GGCATTTTGAACGAGGTCAC AAATATGCGAAGCACCTTGG
*Il13*	GGAGCTGAGCAACATCACACA GGTCCTGTAGATGGCATTGCA
*Il17a*	CCAGGGAGAGCTTCATCTGT ACGTGGAACGGTTGAGGRTAG
*Ifng*	AACGCTACACACTGCATCTTGG GACTTCAAAGAGTCTGAGG
*Ccr3*	TAAAGGACTTAGCAAAATTCACCA TGACCCCAGCTCTTTGATTC
*Mcpt8*	GTGGGAAATCCCAGTGAGAA TCCGAATCCAAGGCATAAAG
*Selp* *(P-selectin)*	AAAAGGTTCCTGGACGCCAA GACGTCATTGAGGTGAGCGA
*Sele* *(E-selectin)*	ACGGATAGAGAGAAGCAGGAGC TCATGAGCTCACTGGAGGCA
*Icam1*	GCTCAGTATCTCCTCCCCA GCTGTGCTTTGAGAACTGTG
*Vcam1*	CCCAAACAGAGGCAGAGTGT CAGGACTGCCCTCCTCTAGT
*Itgb1*	GCTGGGTTTCACTTTGCTGG TGTGCCCACTGCTGACTTAG
*Itgb2*	CAACAACGTCAAGAAGCTGGG GCCTTCTCCTTGTTGGGACA
*Itgb3*	GTGTGGGCCTCAAGATTGGA AGGCACAGTCACAGTCGAAG
*Itgb7*	GACGACTTGGAACGTGTGCG TGGGTGGTGAAGCTTGGAGG
*Itgam*	AAACAAGGATGCTGGGGAGG GTCTCATCAAAGAAGGCACGG
*Itgal*	CTGGACCTGCGTGAAGACC GGTACCGTGGGGCTCCTG
*Itga2b*	AGACACCAGTCAGCTGCTTC CCTGACGGGGCTTCTGTAAG
*Itga4*	TAGCGAATCTTGGCGACATT ACCAACGGCTACATCAACAT
*Itga5*	ATGCCCTGAAGCCAAGTGTT TATTCCCGCTGCAAGAAGGT
*Itgae*	AGCCGGGACATTAACGCCTC ACCACCATGACCTTCAATGCTT

### Histology

Mouse ears were fixed overnight at 4°C in 4% paraformaldehyde and embedded in paraffin. Sections (5 μm) were stained with haematoxylin and eosin.

### Immunohistochemistry staining

For immunohistochemistry (IHC) staining of major basic protein (MBP) and mast cell protease 8 (MCPT8), 5 μm paraffin sections were treated with 0.6% H_2_O_2_ to block endogenous peroxidase activity before antigen retrieval with either Pepsin (Life technologies; for IHC of MBP) or citric buffer (10 mmol/L citric acid, pH 6; for IHC of MCPT8). Slides were then blocked with normal rabbit serum (Vector Laboratories) and incubated overnight with rat anti-mouse MBP (1:2000, provided by Dr James J Lee, Mayo Clinic, Rochester) and rat anti-mouse MCPT8 (1:500, clone TUG8, Biolegend). Slides were then incubated with biotinylated rabbit anti-rat IgG (1:300) and treated with AB complex (Vector Laboratories, Cat No. PK-6104). Staining was finally visualized with AEC high-sensitivity substrate chromogen solution (Dako) and counter-stained with hematoxylin.

### *In vitro* culture of human basophils and quantitative RT-PCR analyses

Human basophils were isolated from the buffy bags of healthy donors (Centre Trinité, L’Établissement Français du Sang, Paris; EFS-INSERM, 18/EFS/041) as previously described (28) by using Basophil Isolation Kit (Miltenyi Biotec, Paris, France). Basophils were then cultured in X-Vivo medium, with 100 ng/0.5 M cells/ml of IL-3, or with 10 nM all-trans RA for 6 hr with or without prior treatment with 1 µM each of retinoic acid receptors (RAR) antagonists CD2665 (RARβ/γ antagonist; Tocris, Cat. 3800) and BMS614 (RARα antagonist; Sigma, Cat. SML-1084) for 1 hr or with RAR antagonists for 1h followed by IL-3 for 6h or with RAR antagonists alone for 1h. Untreated basophils (Baso alone) were used as control.

Total RNA from the different experimental conditions was isolated using the RNeasy minikit (Qiagen, Hilden, Germany). cDNAs were synthesized using a high-capacity cDNA reverse transcription kit (Thermo Fisher Scientific, Courtaboeuf, France), and quantitative PCR was performed with LightCycler 480 (Roche Diagnostics) and QuantiTect SYBR Green Kit (Qiagen) using the primers as described in **Table 3**. Relative RNA levels were calculated with human glyceraldehyde-3-phosphate dehydrogenase (hGAPDH) as an internal control.

**Table 3:** Sequences of PCR primers for human genes.

Human Genes	Sequence 5’ to 3’
*GAPDH*	GTCAAGGCTGAGAACGGGAA AAATGAGCCCCAGCCTTCTC
*ALDH1A2*	TATGTGGATTTGCAGGGCGT ACATCAGCAGGGGGAAGTTC
*ITGB2*	CGACATCATGGACCCCACAA GCATGGAGTAGGAGAGGTCC
*ITGAM*	AGTGCTGGGGGACGTAAATG CCCACTCAGTGACTGACCAA
*ITGA2B*	CTCCTGCTGACTGGCACAC TCAGCCCCTCACTCTGACC
*ITGB7*	ACAGGGGATGCCACAGAATG GCCAGCAGCTCCTCTCGT

### Statistical analysis

Data were analysed using GraphPad Prism 9. Comparison of two groups was performed either by Student’s two-tailed unpaired t-test with Welch’s correction or the two-tailed Mann–Whitney rank sum nonparametric test depending on results from the Kolmogorov–Smirnov test for normality.

## Results

### Basophil accumulation in FITC-induced ACD skin is dependent on adaptive immunity

**Figure 1:**
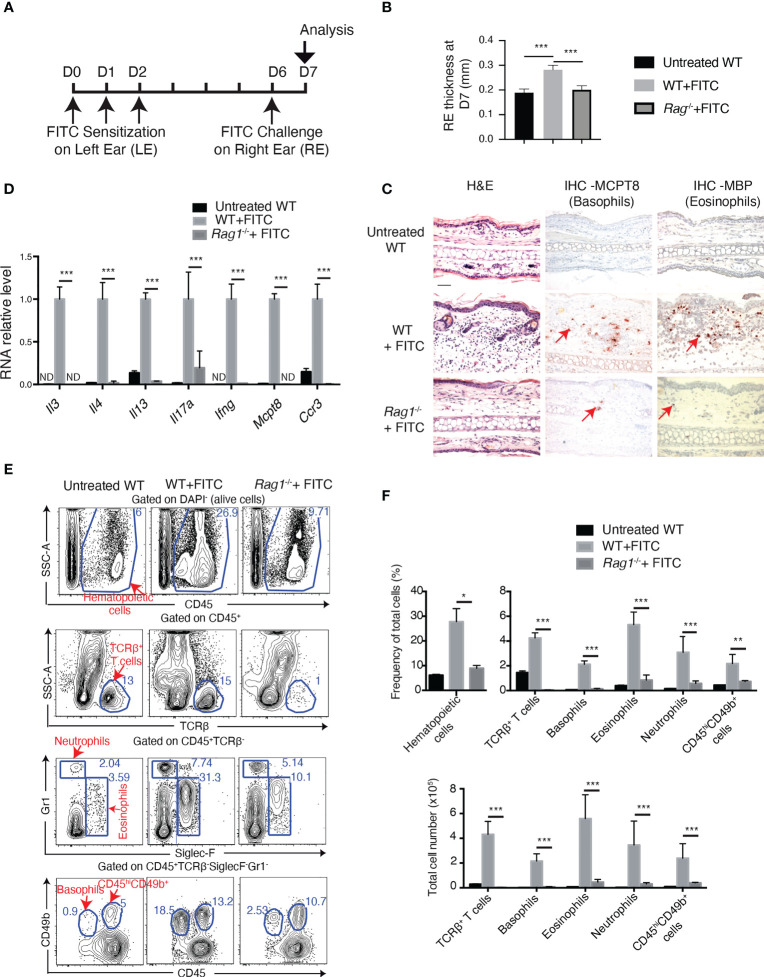
FITC treatment induces basophil accumulation in ACD skin in Rag1-dependent manner. **(A)** Experimental protocol. Eight to twelve-week-old female mice were sensitized with FITC on left ear (LE) at Day **(D)** 0, D1 and D2. Right ears (RE) were then challenged at D6 with FITC and sampled for analyses at D7. **(B)** RE thickness at D7. **(C)** Hematoxylin and eosin (H&E) and immunohistochemistry (IHC) staining of RE sections. Arrow points to one of the positive cells of IHC staining. Scale bar: 50 μm. **(D)** RT-qPCR analyses of cytokines in RE. ND, non-detected. **(E)** FACS analyses of dermal cells of RE for CD45^+^ (hematopoietic cells), CD45^+^TCRβ^+^ T cells, CD45^+^TCRβ^-^Siglec-F^+^Gr1^low-neg^ (eosinophils), and CD45^+^TCRβ^-^Gr1^hi^ (neutrophils), CD45^low^CD49b^+^Siglec-F^-^Gr1^-^(basophils) and CD45^hi^CD49b^+^Siglec-F^-^Gr1^-^ cells (which contain mast cells). **(F)** Comparison of frequency of total cells and total cel numbers. *P≤0.05 **P≤0.01, ***P≤0.001 (Student's t-test). Values are mean ± SEM [**(B)**, n=7; **(D, F)**, n=4 mice per group].

To induce allergic contact dermatitis (ACD) in mice, we employed an experimental protocol (24) in which Balb/c wildtype (WT) mice were first sensitized on one ear (left ear, LE) at Day (D) 0, 1 and 2, with fluorescein isothiocyanate (FITC, a hapten with potential to induce ACD when combined to dibutyl phthalate DBP), and challenged with the same solution on the other ear (right ear, RE) at D6 (**Figure 1A**). This treatment led to an increase in the thickness of RE from FITC-sensitized and challenged mice (**Figure 1B**, compare untreated WT and WT+FITC), but not from mice with only sensitization or only challenge (**Figure S2**). Hematoxylin and eosin (H&E) staining of RE at D7 showed that the FITC treatment induced an inflammatory response with an epidermal hyperplasia and an immune infiltrate in dermis (**Figure 1C**). Immunohistochemistry (IHC) analyses using an antibody against MCPT8 (mast cell protease 8) (29) and an antibody against MBP (major basic protein) (30) revealed the dermal accumulation of basophils and eosinophils, respectively (**Figure 1C**, compare untreated WT and WT+FITC). RT-qPCR analyses showed an increase in RNA levels of cytokines IL-3, IL-4, IL-13, IL-17A and IFN-γ, as well as of MCPT8 (expressed by basophils) and CCR3 (expressed mainly by eosinophils and basophils) in RE from FITC-treated WT compared to untreated WT mice (**Figure 1D**, compare untreated WT and WT+FITC). FACS analyses of dermal cells showed an increased CD45^+^ hematopoietic cells in FITC-treated WT ears compared to untreated ears. These include TCRβ^+^ T cells (identified as CD45^hi^TCRβ^+^), eosinophils (identified as CD45^hi^TCRβ^-^SiglecF^+^), neutrophils (CD45^hi^TCRβ^-^Gr1^hi^), basophils (identified as TCRβ^-^Gr1^-^SiglecF^-^CD45^lo^CD49b^+^), as well as TCRβ^-^Gr1^-^SiglecF^-^CD45^hi^CD49b^+^ cells (which represent a heterogeneous resident cell population containing skin mast cells, called hereafter CD45^hi^CD49b^+^ cells) (**Figures 1E, F**, compare untreated WT and WT+FITC).

It has been reported that in different inflammatory contexts, basophil expansion and accumulation in tissues are adaptive immunity dependent (11, 31–33) or independent (34, 35). To examine whether basophil recruitment in FITC-induced ACD skin is dependent on adaptive immunity, *Rag1^-/-^* mice which lack mature T- and B-lymphocytes were subjected to FITC treatment. Results showed that FITC-induced ACD inflammation was abolished in *Rag1^-/-^* mice, with no increase in RE thickness (**Figure 1B**), largely diminished accumulation of eosinophils, basophils, neutrophils and CD45^hi^CD49b^+^ cells (which contain mast cells) (**Figures 1C, E, F**), and no increase in cytokine expression (**Figure 1D**). These results thus indicate that skin recruitment of basophils, as well as other immune cells in FITC-induced ACD are dependent on adaptive immunity.

### IL-3 is crucial for basophil accumulation in FITC-induced ACD skin

**Figure 2:**
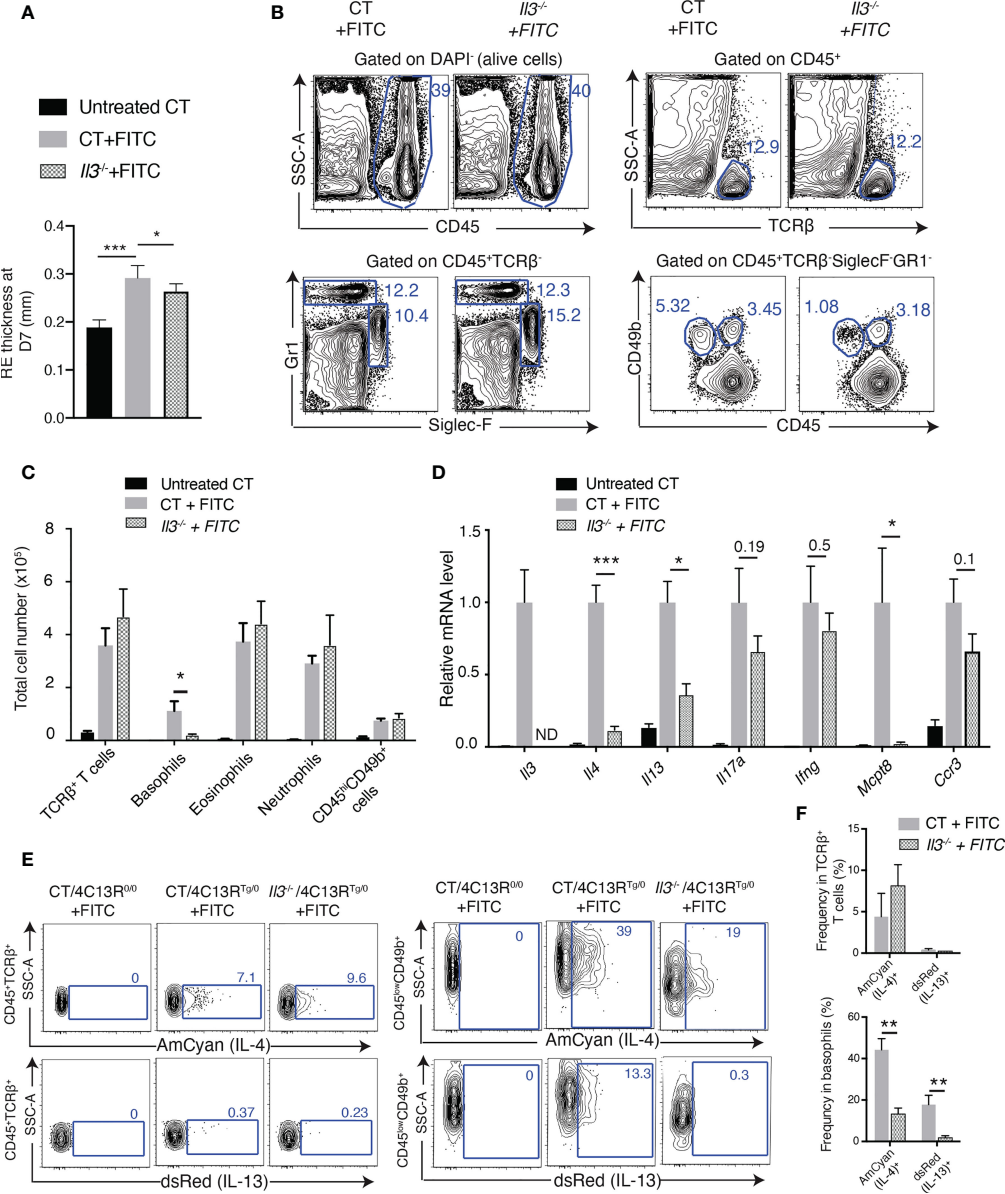
IL-3 plays a crucial role for basophil recruitment to FITC-induced ACD skin. **(A)** Thick- ness of FITC-challenged right ear (RE) at D7. **(B)** FACS analyses of dermal cells of RES from FITC-treated 113 and wildtype control (CT) littermate mice, for CD45^+^ hematopoietic cells, CD45^+^TCRβ^+^ T cells, CD45^+^TCRβ^-^Siglec-F^+^Gr1^low-neg^ (eosinophils), and CD45^+^TCRβ^-^Gr1^hi^ (neutrophils), CD45^low^CD49b^+^TCRβ^-^Siglec-F^-^Gr1^-^ (baso- phils) and CD45^hi^CD49b^+^TCRβ^-^Siglec-F^-^Gr1^-^ cells (which contain mast cells),. **(C)** Comparison of total cell numbers in RE. **(D)** RT-qPCR analyses of REs. ND, non detectable. **(E)** FACS analyses of Amcyan (IL-4) and dsRed (IL-13) expression by CD45^+^TCRβ^+^ T cells (left panel) and by CD45^low^CD49b^+^ basophils (right panel), in the dermis of FITC-treated CT/4C13R^Tg/0^ and *Il3* ^-/-^ */*4C13R^Tg/0^ mice. FITC-treated CT/4C13R^0/0^ was used to set the gating for AmCyan and dsRed. **(F)** Comparison of frequencies of AmCyan (IL-4)^+^ cells and dsRed(IL-13)^+^ cells in TCRβ^+^ T cells or in basophils. *P≤0.05 **P≤0.01, ***P<0.001 (Student's t-test). Values are mean ± SEM [**(A)**, n=7; **(C, D, F)**, n=4 mice per group].

**Figure 3:**
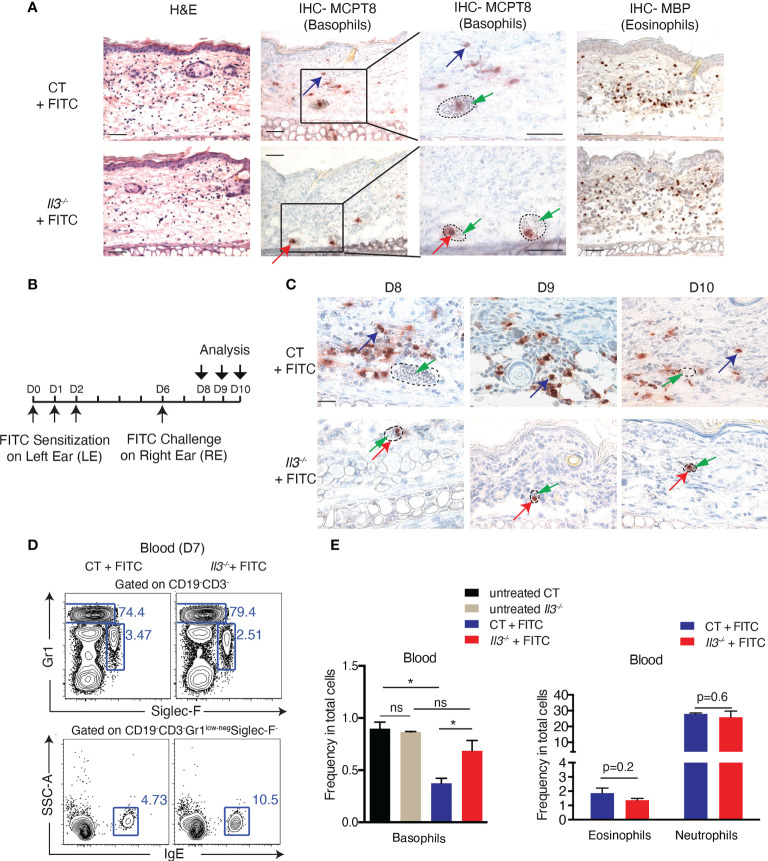
IL-3 is crucial for basophil extravasation to FITC-induced ACD skin. **(A)** HE and IHC staining of RE sections at D7. Red arrow points to one of basophils inside blood vessels, whereas blue arrow points to one of basophils out of blood vessels. The green arrow points to red blood cells inside the vessel. Dashed circles outline blood vessels. **(B-C)** *Il3* ^-/-^ and CT mice were sensitized at D0, D1 and D2 on LEs and challenged at D6 on REs, which were analysed at D8, D9 and D10 **(B)** for Mcpt8 IHC staining **(C)**. Scale bar, 50 μm. **(D)** Representative FACS plots of blood cells for CD19^-^CD3^-^Gr1^-^SiglecF^-^IgE^+^ (basophils), CD19^-^CD3^-^Siglec-F^+^Gr1^low-neg^ (eosinophils), and CD19^-^CD3^-^Gr1^hi^ (neutrophils) from FITC-treated CT and *Il3* ^-/-^ mice. **(E)** Comparison of frequency of basophils (left panel), eosinophils and neutrophils (right panel) in total cells. *P≤0.05 (Student's t-test). ns, non significant. Values are mean ± SEM (n≥3 mice per group).

Based on the above observation that IL-3 expression in ACD skin was totally abolished in *Rag1* ^-/-^ mice, we next examined the role of IL-3 in ACD skin inflammation. *Il3^-/-^* and their wildtype control (CT) littermate mice were subjected to the FITC treatment. Measurement of RE thickness showed a modest but significant decrease in FITC-treated *Il3* ^-/-^ mice compared to FITC-treated WT mice (**Figure 2A**). FACS analyses showed that the number of basophils was highly reduced in RE from FITC-treated *Il3^-/-^* mice compared to that from FITC-treated CT mice (**Figures 2B, C**), which was also confirmed by IHC staining for basophils and eosinophils (see **Figure 3A**). In contrast, no decrease was observed in the number of TCRβ^+^ T cells, eosinophils, neutrophils or CD45^hi^CD49b^+^ cells (which contain mast cells) (**Figures 2B, C**), indicating a specialized requirement of IL-3 for basophil recruitment and accumulation in ACD skin.

In addition, RT-qPCR analyses of RE showed that RNA levels of MCPT8, IL-4 and IL-13 were significantly decreased in FITC-treated *Il3* ^-/-^ mice compared to FITC-treated CT mice (**Figure 2D**). As IL-4 and IL-13 have been reported to be produced by various cell types including Th2 cells and basophils (36), we further investigated the cells in which IL-4 and IL-13 expression was reduced in FITC-treated *Il3^-/-^* skin. For this purpose, we bred *Il3^-/-^* mice with *4C13R* dual reporter mice (which have transgenic expression of the cyan fluorescent protein AmCyan under the control of Il4 regulatory elements and the red fluorescent protein dsRed under the control of Il13 regulatory elements) (37). FACS analyses of RE showed that first, IL-4 (AmCyan) and IL-13 (dsRed) were detected in both TCRβ^+^ T cells and basophils in FITC-treated CT/4C13R^Tg/0^ mice (**Figures 2E, F**); second, AmCyan (IL-4) and dsRed (IL-13) expression in TCRβ^+^ T cells was comparable between FITC-treated CT/4C13R^Tg/0^ and *Il3^-/-^*/4C13R^Tg/0^ skin (**Figures 2E, F**). In contrast, their expression in basophils was diminished in FITC-treated *Il3^-/-^*/4C13R^Tg/0^ skin (**Figures 2E, F**), indicating that basophils but not Th2 cells were responsible for the reduction of IL-4 and IL-13 expression detected in RE from FITC-treated *Il3* ^-/-^ mice.

Together, these results suggested that in ACD skin, IL-3 was specifically and crucially required for the accumulation of basophils, which were the major cell type contributing to the induced expression of Th2 cytokines IL-4 and IL-13 in FITC-induced ACD.

### IL-3 is crucial for basophil extravasation to ACD skin

To examine basophils in FITC-treated WT and *Il3^-/-^* skin in histological level, we performed MCPT8 IHC staining. Of interest, we observed that in addition to the decrease in basophil number, all the detected basophils were strikingly restricted inside blood vessels in RE of FITC-treated *Il3^-/-^* mice (**Figure 3A**; MCPT8-labled basophils were immersed in red blood cells), indicating a defect in basophils for crossing the vascular endothelium. In contrast to basophils, no difference was observed in eosinophil extravasation to skin between FITC-treated *Il3^-/-^* and CT mice (**Figure 3A**).

To examine whether this observation could reflect a delayed basophil recruitment in *Il3^-/-^* mice, we performed FITC treatment and analysed RE at later time points (D8, D9 and D10) (**Figure 3B**). Similar phenotype (restriction of basophils inside blood vessels in the skin) was observed as at D7, indicating that basophils in FITC-treated *Il3^-/-^* mice were not able to cross vascular endothelium at any of these time points (**Figure 3C**). Thus, basophil extravasation was not delayed but defective in FITC-treated *Il3^-/-^* mice.

Next, we performed FACS analyses of blood basophils, which were identified as CD19^-^CD3^-^Gr1^-^Siglec-F^-^IgE^+^ cells (**Figure 3D**). Blood eosinophils and neutrophils were identified as CD19^-^CD3^-^ Siglec-F^+^Gr1^low-neg^, CD19^-^CD3^-^Gr1^hi^, respectively (**Figure 3D**). Results showed that first, the frequency of basophils in the blood was comparable between untreated CT and *Il3^-/-^* mice (**Figure 3E**, compare untreated CT with untreated *Il3* ^-/-^), indicating that IL-3 was not necessary for the development of baseline levels of basophils in mice, in agreement with previous reports (9, 38, 39); second, the frequency of basophils in the blood of FITC-treated CT mice was lower compared to untreated CT mice (**Figure 3E**, compare CT+FITC with untreated CT), likely due to the skin recruitment of basophils; and third, such decrease was not observed in FITC-treated *Il3^-/-^* mice (**Figure 3E**, compare *Il3^-/-^* + FITC with untreated *Il3^-/-^*), which was fitting with the observation that basophils were not able to cross vascular endothelium to enter the skin in these mice. In contrast to basophils, no difference was observed for frequency of eosinophils and neutrophils in the blood between FITC-treated *Il3^-/-^* and CT mice (**Figure 3E**). Altogether, these data suggested that basophil extravasation to inflamed ACD skin was defective in mice lacking IL-3.

### IL-3 produced by T cells mediates basophil extravasation to ACD skin

**Figure 4:**
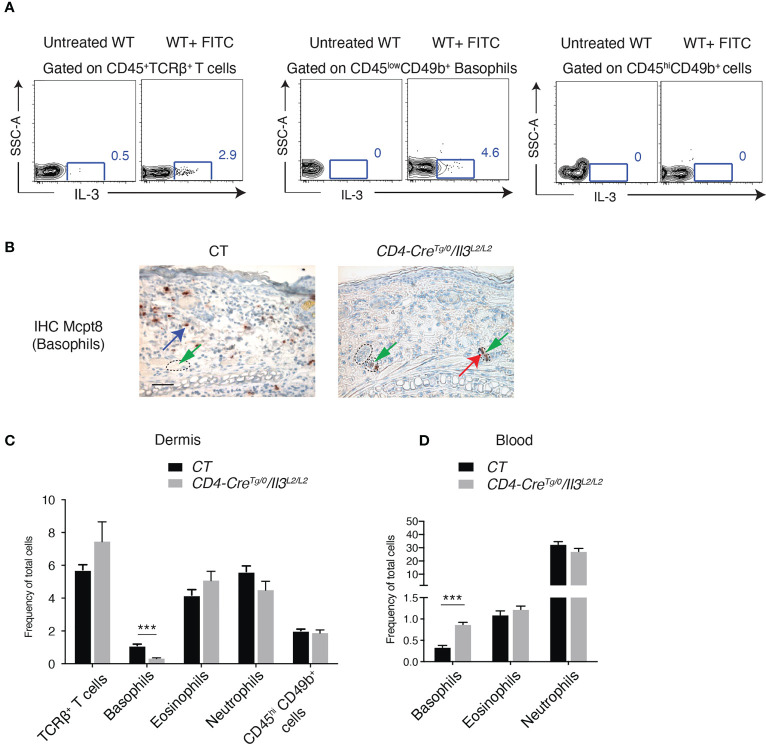
IL-3 produced by T cells mediates basophil extravasation to FITC-induced ACD skin. **(A)** Intracellular staining of IL-3 in dermal cells of RES from untreated or FITC-treated Balb/c WT mice. **(B)** IHC staining with Mcpt8 antibody in RE of FITC-treated CD4-Cre^0/0^/*Il3* ^L2/L2^ (CT) and CD4-Cre^Tg/0^/*Il3* ^L2/L2^ mice. Red arrow points to one of basophils inside blood vessels and blue arrow points to one of basohils of our blood vessel. Dashed circles outline blood vessels. Scale bar, 50μm. **(C)** FACS analyses of dermal cells of REs from FITC-treated CT and CD4-Cre^Tg/0^/*Il3* ^L2/L2^ mice, for CD45^+^ hematopoietic cells, CD45^+^TCRβ^+^ T cells, CD45^+^TCRβ- Siglec-F^+^Gr1^low-neg^ (eosinophils), and CD45^+^TCRβ^-^Gr1^hi^ (neutrophils), CD45^low^CD49b^+^Siglec-F^-^Gr1^-^(basophils) and CD45^hi^CD49b^+^Siglec-F^-^Gr1^-^ cells (which contain mast cells). **(D)** FACS analyses of blood cells from FITC-treated CT and CD4-Cre^Tg/0^/*Il3* ^L2/L2^ mice for CD19^-^CD3^-^Gr1^low-neg^SiglecF^-^IgE^+^ (basophils), CD19^-^CD3^-^Siglec-F^+^Gr1^low-neg^ (eosinophils), and CD19^-^CD3^-^Gr1^hi^ (neutrophils). ***P≤0.001 (Student's t-test). Values are mean ± SEM (n=4 mice per group).

By performing intracellular staining, we showed that IL-3 was detected in both TCRβ^+^ T cells and basophils of FITC-treated WT skin (**Figure 4A**). To examine whether IL-3 produced by T cells mediates basophil recruitment to ACD skin, we generated mice in which IL-3 is ablated selectively in both CD4^+^TCRβ^+^ and CD8^+^TCRβ^+^ T cells, by breeding *Il3^L2/L2^* with *CD4-Cre^Tg/0^* mice (25). Results showed that similar to what was observed in FITC-treated *Il3^-/-^* skin, all basophils detected by IHC-MCPT8 were confined inside blood vessels (**Figure 4B**). FACS analysis of FITC-challenged *CD4-Cre^Tg/0^/Il3^L2/L2^* skin showed a diminished frequency of basophils, while no decrease was observed in TCRβ^+^ T cells, eosinophils, neutrophils or CD45^hi^CD49b^+^ cells (which contain mast cells) (**Figure 4C**). In addition, a higher frequency of basophils in blood was seen in FITC-treated *CD4-Cre^Tg/0^/Il3^L2/L2^* mice compared to FITC-treated wildtype CT mice (**Figure 4D**), again suggesting a defective extravasation of basophils to ACD skin in these mice. Together, these results indicated that IL-3 produced by T cells was crucial for basophil extravasation in ACD skin.

### Decreased expression of integrins in basophils from FITC-treated *Il3^-/-^* skin

It has been recognized that leukocyte extravasation is regulated by a concerted multistep actions between leukocytes and endothelial cells (ECs) including rolling, adhesion and TEM (13). IL-3 receptor was previously shown to be expressed by both human ECs (19, 20) and human/mouse basophils (40, 41). *In vitro* studies have suggested that basophils or ECs could respond to IL-3 signalling: IL-3 stimulation of human basophils enhances their adhesiveness to ECs (23) and their TEM (22); on the other hand, stimulation of human ECs by IL-3 induced the expression of P-selectin and selective basophil accumulation (21, 42).

**Figure 5:**
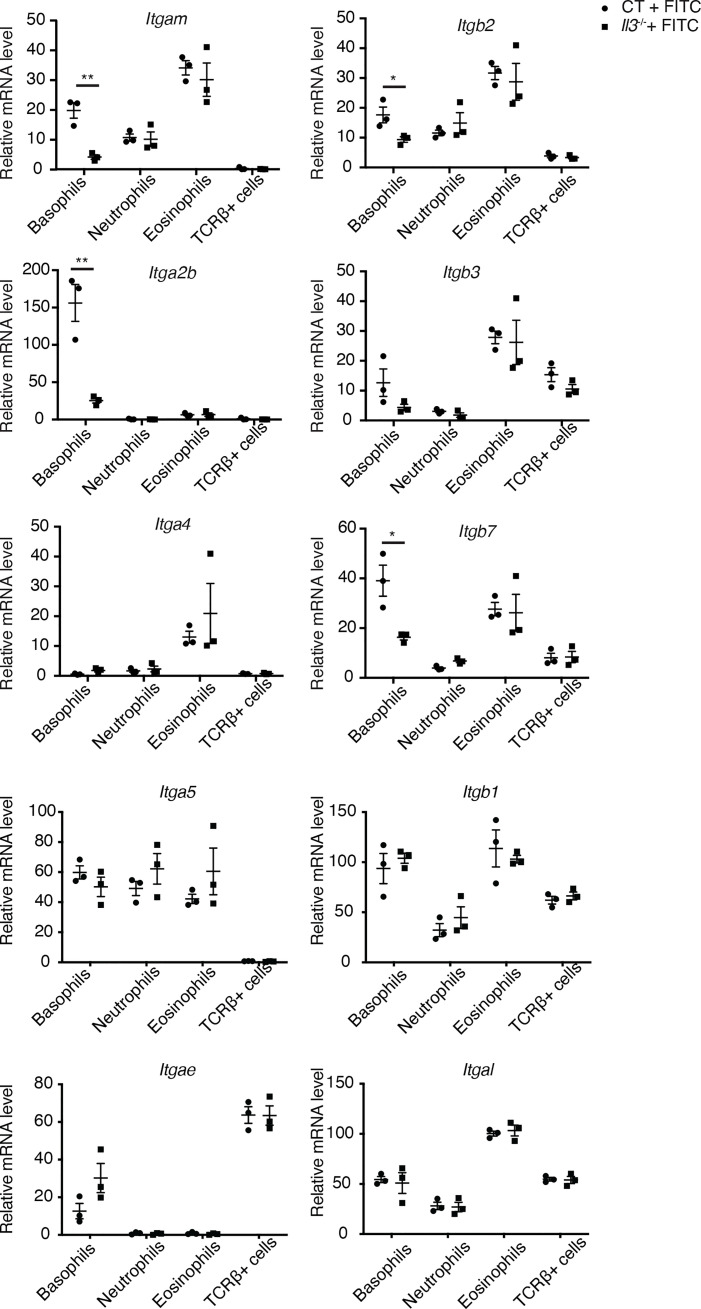
Decreased expression of integrins by basophils sorted from FITC-treated *Il3* ^-/-^ skin. RT-qPCR analyses of the sorted basophils, neutrophils, eosinophils and TCRβ^+^ T cells. CT, wildtype controls. *P≤0.05 **P≤0.01 (Student's t-test). Values are mean ± SEM (n=3 mice per group).

We thus sorted ECs and basophils by FACS from FITC-challenged mouse RE and analysed by RT-qPCR the expression of molecules potentially implicated in basophil-EC interaction. First, the expression of *Selp* (P-selectin), *Sele* (E-selectin), *Icam1* and *Vcam1* was much higher in ECs compared to CD45^+^ hematopoietic cells, however, no decrease in these genes was observed in ECs from *Il3^-/-^* compared to CT mice (**Figure S3**). On the other hand, analyses of the sorted basophils (note that basophils sorted from the FITC-treated *Il3^-/-^* RE corresponded to those stuck inside blood vessels) revealed a significant decrease in *Itgam*, *Itgb2*, *Itga2b* and *Itgb7* from FITC-treated *Il3^-/-^* compared to CT mice (**Figure 5**), whereas no significant difference was observed for *Itga4*, *Itga5*, *Itgae*, *Itgb1*, *Itgal* and *Itgb3* (**Figure 5**). Importantly, the decrease in *Itgam*, *Itgb2*, *Itga2b* and *Itgb7* was specific for basophils, as no change was observed for neutrophils, eosinophils or TCRβ^+^ T cells from FITC-treated *Il3^-/-^* compared to CT mice (**Figure 5**). It is also notable that these genes were all highly expressed in basophils compared to neutrophils, eosinophils and TCRβ^+^ T cells from FITC-treated CT mice (**Figure 5**). Together, these data revealed an IL-3-dependent expression of integrins ITGAM, ITGB2, ITGA2B and ITGB7 in basophils, which are potentially implicated in basophil-EC interaction during the extravasation process in FITC-induced ACD.

### Retinoic acid signaling promotes basophil extravasation to ACD skin

**Figure 6:**
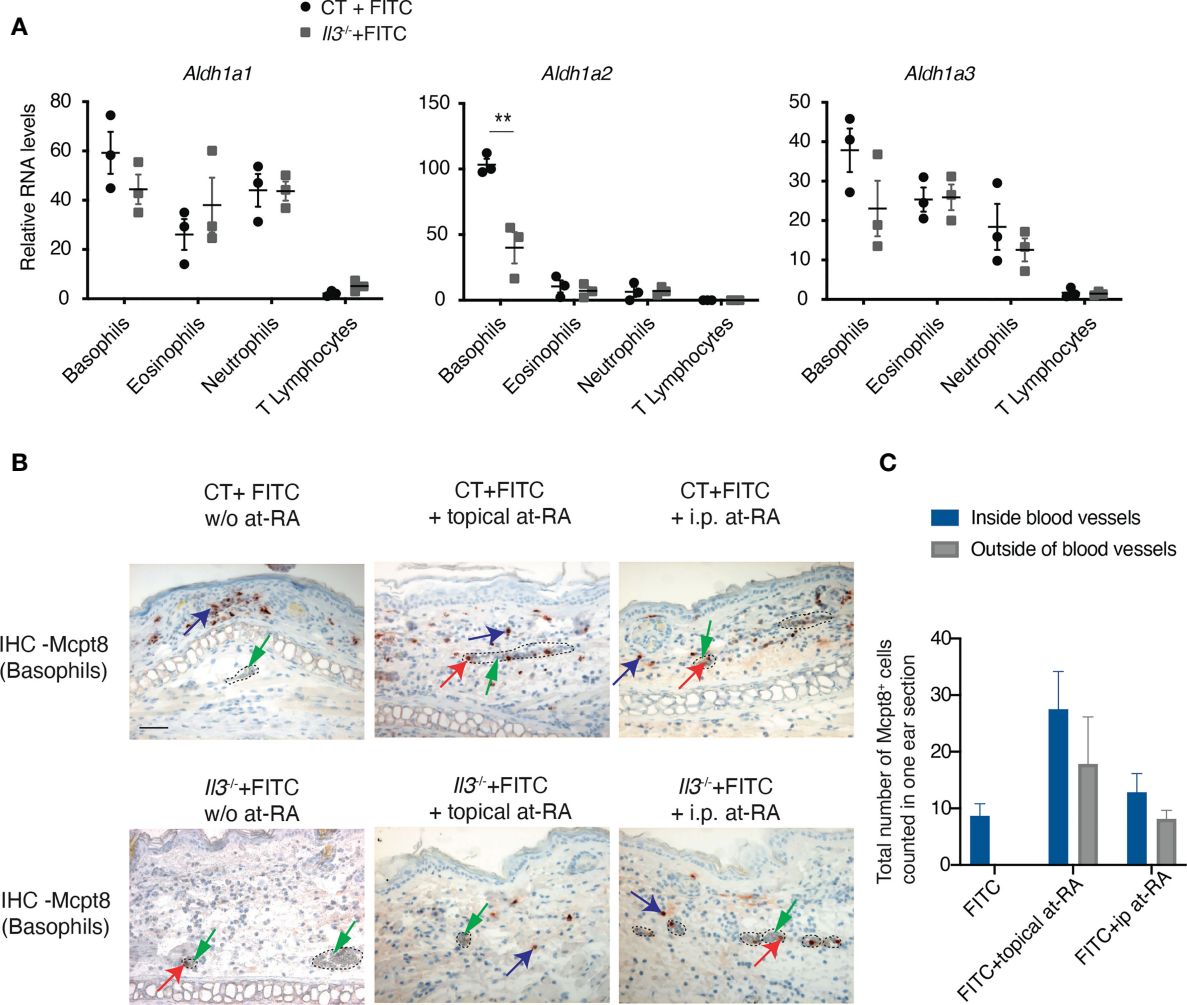
All-trans retinoic acid treatment prior to FITC challenge promotes basophil recruitment to ACD skin. **(A)** RT-qPCR analyses of retinaldehyde dehydrogenease genes Aldh1a1, Aldh1a2, and Aldh1a3 in basophils, neutrophils, eosinophils and T cells sorted from FITC-treated *Il3* ^-/-^ and CT ears. **P≤0.01 (Student's t-test). Values are mean ± SEM (n=3). **(B)** *Il3* ^-/-^ and CT mice were FITC-sensitized and -challenged as described in **Figure 1A**. Mice were either topically treated with all-trans retinoic acid (at-RA) on RE at 2 hr before FITC-challenge, or intraperitoneally (i.p.) administrated with at-RA at 24 hr before FITC-challenge. RE sections from *Il3* ^-/-^ and CT mice were used for MCPT8 IHC. Blue arrows point to one of the positive cells outside of blood vessels; red arrows point to one of the positive cells inside blood vessels; green arrows point to red blood cells inside the vessel. Dashed circles outline blood vessels. Scale bar: 50 μm. **(C)** Comparison of total number of Mcpt8 basophils inside and ourside of blood vessels from ear sections of *Il3* ^-/-^ mice treated with FITC, FITC+topical at-RA, or FITC+i.p. at-RA. Values are mean ± SEM (n=6).

We next sought to explore how IL-3 signalling regulates the expression of integrins by basophils. Of interest, it was previously reported that in human basophils co-cultured with mast cells, mast cell-derived IL-3 induces the expression of the retinaldehyde dehydrogenease ALDH1A2 (also called RALDH2), an enzyme that catalyses the last oxidative step of the cascade leading retinol to produce retinoic acid (RA) (43). It was shown that RA produced by basophils promotes the expression of ITGA4/ITGB7 heterodimer on T cells in a paracrine manner, thus influencing T cell polarisation (43). Other studies reported the induction of ALDH1A2 in human basophils (44) or ALDH1A3 (also called RALDH3) in mouse basophils (45) upon the stimulation of IL-3 and IL-33/IgE stimulation, respectively. We then examined the expression of *Aldh1a1*, *Aldh1a2* and *Aldh1a3* in basophils, eosinophils, neutrophils and TCRβ^+^ T cells sorted by FACS from FITC-treated WT and *Il3^-/-^* RE. Results show that RNA levels for *Aldh1a2*, but not *Aldh1a1* or *Aldh1a3*, were significantly decreased in basophils from FITC-treated *Il3^-/-^* compared to CT mice (**Figure 6A**), whereas its levels in eosinophils, neutrophils or TCRβ^+^ T cells were all low and remained unchanged between FITC-treated *Il3^-/-^* and CT mice (**Figure 6A**). These results thus suggested that *Aldh1a2* expression by basophils from FITC-treated skin is dependent on IL-3. Notably, RT-qPCR analyses of naïve basophils sorted from spleen in steady state showed that *Aldh1a2* was undetectable (qPCR cross point >50) in basophils from both wildtype control (CT) and *Il3* ^-/-^ mice (**Figure S4**). In addition, RT-qPCR analyses showed that except for *Itgam*, which was significantly lower in basophils from *Il3* ^-/-^ mice compared to CT mice, the other ITGs analyzed including *Itgb2*, *Itga2b*, *Itgb3*, *Itgae* and *Itgb7* (a slight tendency; p=0.07) did not exhibit significant difference between basophils from CT and *Il3* ^-/-^ mice (**Figure S4**). These results thus suggested that in contrast to the inflamed context where IL-3 played a significant role in regulating the RNA expression of *Aldh1a2* and ITGs, in steady state, IL-3-ALDH1A2 axis was minimally implicated in regulating the expression of ITGs in basophils.

We further tested whether RA administration restores basophil extravasation in *Il3^-/-^* mice. Wildtype CT and *Il3^-/-^* mice were treated with FITC as described in **Figure 1A**, and all-trans RA (at-RA) was either topically applied to RE 2 h before the FITC-challenge, or injected i.p. 24 h before the FITC-challenge. Results show that upon at-RA topical treatment, more basophils were accumulated in FITC-treated CT skin (**Figure 6B**, compare CT +FITC w/o at-RA and CT +FITC + topical at-RA). Moreover, while basophils were stuck inside blood vessels in FITC-treated *Il3^-/-^* (w/o at-RA) mice, at-RA topical treatment resulted in more basophils detected outside the blood vessels (**Figures 6B, C**). Similarly, i.p. injection of at-RA led to an increased number of basophils outside the blood vessels of FITC-treated *Il3^-/-^* skin, although such effect appeared to be relatively weaker compared to topical RA treatment (**Figures 6B, C**). Taken together, these data suggested that the administration of at-RA has an effect to restore basophil extravasation in *Il3^-/-^* mice.

### IL-3 stimulation of human basophils upregulates integrin particularly ITGB7 in RA signaling-dependent manner

**Figure 7:**
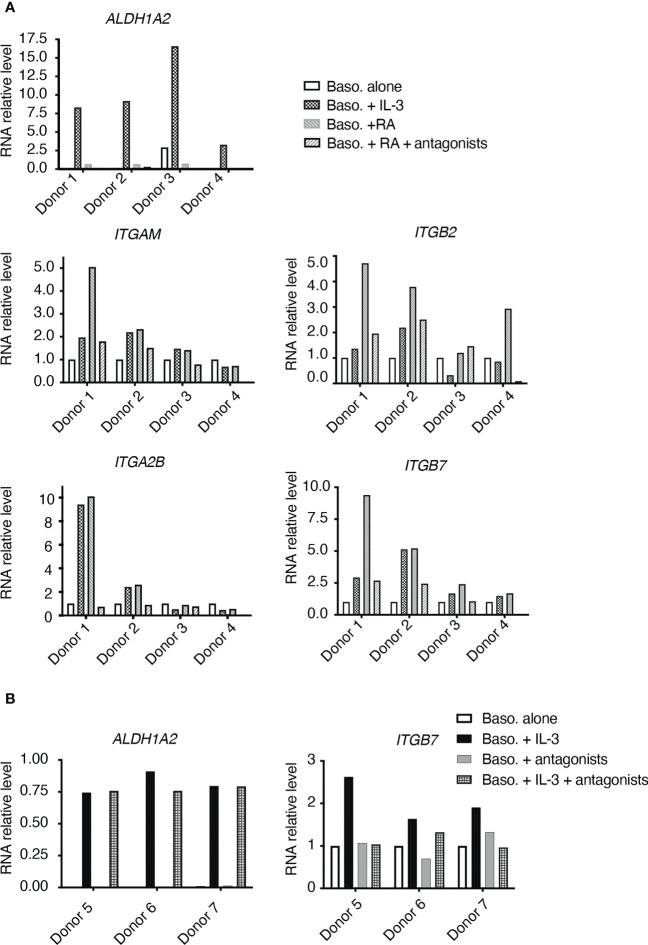
RT-qPCR analyses of in vitro cultured human basophils. **(A)** Basophils isolated from 4 donors (Donor 1, 2, 3, 4) were cultured alone, or in the presence of IL-3, all-trans retinoic acid (RA), or RA+RAR antagonists. **(B)** Basophils isolated from 3 donors (Donor 5, 6, 7) were cultured alone, or in the presence of IL-3, RAR antagonist, or IL-3+RAR antagonists.

To examine the human relevance of the above findings in mouse, we performed *in vitro* culture of human primary basophils isolated from healthy donors. We first confirmed that *ALDH1A2* expression was highly induced by IL-3 in basophils particularly from Donor 1, 2 and 3, while the Donor 4 exhibited relatively less induction of *ALDH1A2* (**Figure 7A**). Further examination of integrin expression showed that basophils from Donor 1 and Donor 2 showed an increased expression of *ITGAM*, *ITGB2*, *ITGA2B* and *ITGB7* upon IL-3 stimulation (**Figure 7A**), while the induction of aforementioned integrins was less clear in the basophils from Donor 3 and Donor 4 (**Figure 7A**). Moreover, when stimulated with RA, basophils from Donor 1 and Donor 2 also showed an increased expression of these integrins, which was reduced upon the treatment with RAR antagonists (**Figure 7A**). Particularly, *ITGB7* expression was increased in basophils from all the 3 donors (Donor 1-3) upon RA stimulation, which was antagonized by RAR antagonists (**Figure 7A**). Though Donor 4 did not show a dramatic increase in ITGB7 expression, its expression was completely antagonized by RAR antagonists.

In another set of experiment, basophils were treated with IL-3 plus RAR antagonists. As shown in **Figure 7B**, *ALDH1A2* expression was induced by IL-3 and was not affected by the addition of RAR antagonists. *ITGB7* expression was induced by IL-3, and such induction was suppressed by the addition of RAR antagonists (**Figure 7B**). These data thus suggested that IL-3 stimulation upregulates ITGB7 expression in human basophils in an RA signaling-dependent manner.

## Discussion

In this study, we investigated basophil recruitment to allergic skin with a hapten-induced ACD mouse model. Making use of our newly generated IL-3-knockout and conditional knockout mouse lines, our data demonstrated a crucial role for IL-3 produced by T cells in mediating basophil extravasation to the inflamed skin. Moreover, we found that basophils from FITC-treated IL-3-knockout mice had a decreased expression of several integrins including *Itgam*, *Itgb2*, *Itga2b* and *Itgb7*, which was associated with the failure of basophils in crossing ECs to enter inflamed skin site of these mice. Interestingly, basophils from FITC-treated IL-3-knockout mice exhibited a reduced expression of *Aldh1a2*, and administration of at-RA restored basophils extravasation in these mice. Finally, we show that as observed in mice, human primary basophils express *ALDH1A2* upon IL-3 stimulation, and that IL-3-induced expression of integrins particularly *ITGB7* was dependent on RA signaling.

Our data point to a central role of IL-3 in basophil extravasation into the inflamed ACD skin, which involves a cooperation between T cells, basophils and ECs. Yet, it remains to be determined when and how IL-3 is induced in CD4^+^ T cells upon the sensitization and challenge. Though we show that IL-3-expressing TCRβ^+^ T cells are accumulated in RE from FITC-treated mice but not from untreated mice, thus suggesting that IL-3 at the challenge phase is likely responsible for its effect on basophil extravasation, it does not exclude a possible role of IL-3 during the sensitization phase of ACD. To explore this, temporal knockout of IL-3 (e.g. using tamoxifen-inducible Cre-ER^T2^ system), or blockade of IL-3 signaling using neuralizing antibody or antagonists to IL-3 or IL-3Rα (IL-3 specific receptor subunit), during sensitization or challenge phase would be useful. This will also provide information on the appropriate time window to target IL-3 axis and to test their effects on blocking the recruitment of basophils to inflamed skin, thereby modulating the established inflammation.

While IL-3 could exert multiple functions, our data revealed that IL-3 signalling on basophils was crucial for these cells to upregulate their RNA expression of integrins including *Itgam*, *Itgb2*, *Itga2b* and *Itgb7*. Indeed, the upregulation of *Itgam* was previously reported for mouse basophils stimulated *in vitro* with IL-3 (34), and for human basophils (23), which enhances their adhesiveness to ECs. In addition, *in vitro* studies suggested that IL-3 could stimulate human basophil rolling and adhesion to ECs, and blocking Abs against ITGB1, ITGB2, ITGAM and ITGAL inhibited basophil rolling and adhesion to ECs (21–23). Here, our data identified that in addition to *Itgam* and *Itgb2* as previously reported, *Itgb7* and *Itga2b* were also regulated by IL-3 signaling. Particularly, *Itgb7* and *Itga2b* are highly expressed by basophils compared neutrophils, eosinophils and T cells from FITC-treated wildtype mice (**Figure 4**), and moreover, in our tests with human primary basophils stimulated with IL-3, *ITGB7* induction was most reproducible, suggesting a potential role of ITGB7 in basophil extravasation, which deserves further investigation.

Our data suggest a possible IL-3-RA axis through ALDH1A2 expression to regulate the gene expression of integrins in basophils. First, we showed that in FITC-treated mice, *Aldh1a2* expression by basophils is diminished in IL-3-KO mice, while in human primary basophils, IL-3 stimulation induces *ALDH1A2* expression, suggesting a conserved regulation of ALDH1A2 by IL-3 from mouse to human. These data are in good agreement with previous reports, which show that IL-3 induces ALDH1A2 expression and RA production by basophils (43, 44). It should be noted that genetic polymorphism in human IL-3Rα has been documented (46, 47), and our previous data have also revealed variations among healthy donors in their response to IL-3 (48), thus pointing towards polymorphism in IL-3Rα as one potential factor, which determines response of basophils to IL-3 and as a consequence, induction of ALDH1A2. This could explain the difference in the induction of ALDH1A2 in IL-3 stimulated basophils from different donors in our human experiment (**Figure 7A**; donor 4 had much lower ALDHL1A2 expression compared with other 3 donors). In addition, genetic polymorphism of RAR/RXR (receptors for RA) or RA response elements can impact the transcriptional regulation of ITGs by RA signaling, which may also explain the differential induction of ITG in IL-3-stimulated basophils among the donors (**Figure 7A**). Interestingly, it was previously proposed that RA produced by basophils promotes the expression of ITGA4/ITGB7 heterodimer on T cells in a paracrine manner thus influencing T cell polarisation (43). In contrast, our study provides evidence that RA promotes the expression of integrins particularly *ITGB7* in human basophils, and IL-3-induced *ITGB7* could be suppressed by RAR antagonists. Moreover, at-RA administration could restore at least partially basophil extravasation to the skin in *Il3* ^-/-^ mice. Thus, RA produced by basophils may act in an autocrine manner to regulate the expression of integrins implicated in basophil extravasation.

**Figure 8:**
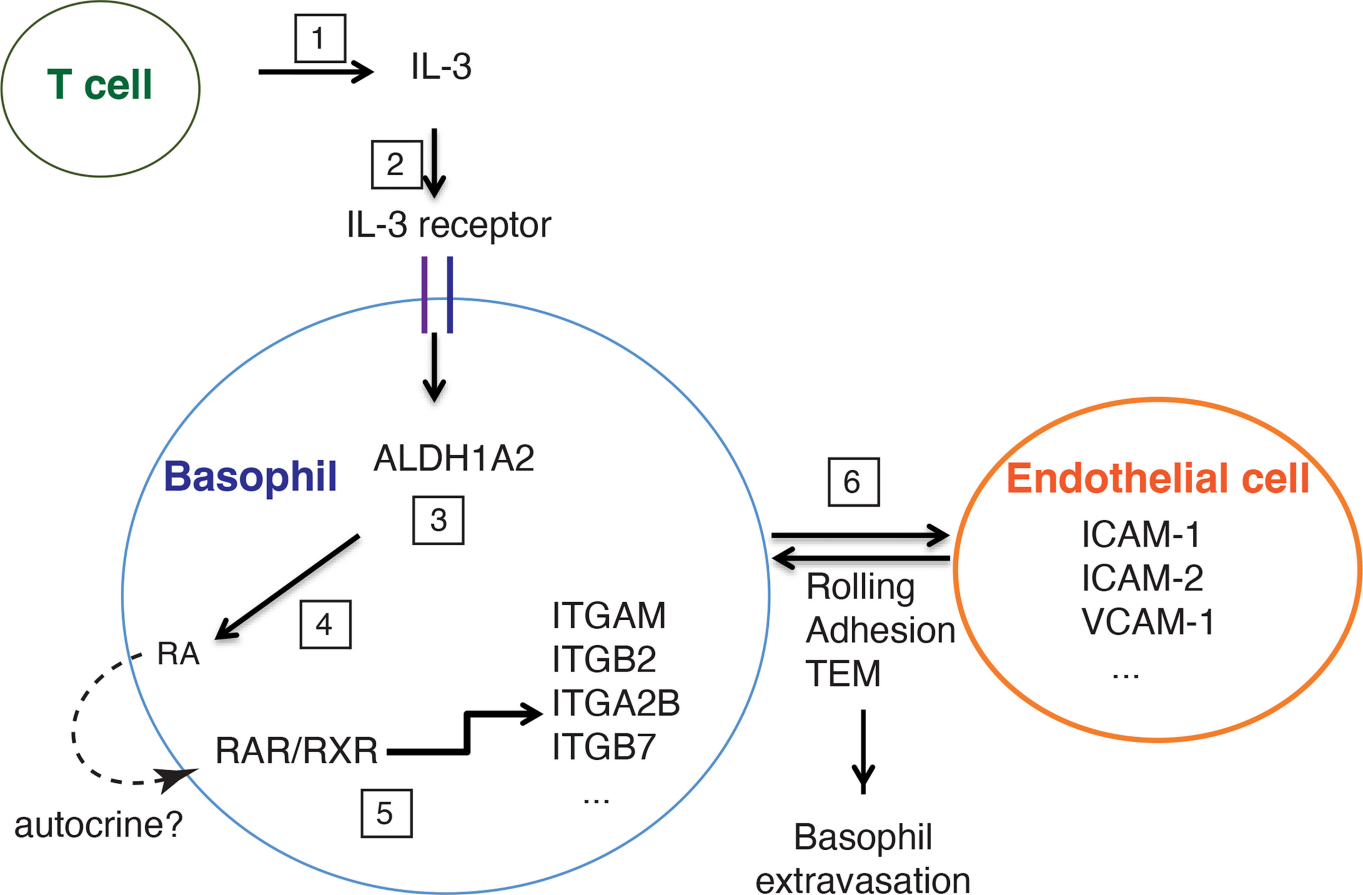
A schematic representation of the role of T cell-derived IL-3 in mediating FITC-induced basophil extravasation to ACD skin. Upon FITC treatment, T cells secrete IL-3 (1), which binds to IL-3 receptor complex on basophils (2), and induces expression of ALDH1A2 (3), leading to the production of retinoic acid (RA) by basophils (4). In turn, RA activates RAR/RXR receptor heterodimer in an autocrine manner, which upregulates the expression of integrins such as ITGAM, ITGB2, ITGA2B, ITGB7 by basophils (5). The interaction between integrins (expressed by basophils) and their ligands (expressed by endothelial cells, such as ICAM-1, ICAM-2, VCAM-1) is crucial for baso- phil extravasation to ACD skin through rolling, adhesion and trans-endothelial migration (TEM) (6).

Based on these data, we propose a model illustrated in **Figure 8**: upon hapten sensitization and challenge, T cells secrete IL-3, which binds to IL-3 receptor complex on basophils and induces expression of ALDH1A2, resulting in the production of RA by basophils; in turn, RA activates RAR/RXR receptor heterodimer in basophils in an autocrine manner, and thereby upregulates the expression of integrins ITGAM, ITGB2, ITGA2B, and ITGB7, promotes the interaction between basophils and ECs, and eventually permits basophil extravasation to ACD skin. To fully determine the role of IL-3-RA axis in basophil extravasation process, mice in which *Aldh1a2* is conditionally knocked out in basophils (breeding *Aldh1a2* ^L2/L2^ mice with *Mcpt8* ^Cre^) will be useful to provide evidence on whether this enzyme and RA production are crucial for basophil extravasation. One might also envisage to use RARE (RAR responding element) reporter mice to track RA production and activity in basophils during inflammatory processes. Moreover, it will be also interesting to test whether RAR antagonists could block basophil recruitment to inflamed skin site. We could not provide data at this stage with the *in vivo* administration of RAR antagonists (CD2665 and BMS614) due to their toxicity in mice (data not shown), but further investigation on the possible strategies to block RA synthesis and signaling in basophils, as well as to target key molecular players including integrins (e.g. using blockade antibodies), should be further tested. Finally, it will be interesting to examine whether the proposed mechanism applies generally to basophil-related skin pathologies (1), such as allergen (e.g. house dust mite)-induced atopic dermatitis or urticaria, which will help to develop strategies for treating these diseases.

## Data availability statement

The raw data supporting the conclusions of this article will be made available by the authors, without undue reservation.

## Ethics statement

The studies involving human participants were reviewed and approved (18/EFS/041) by the ethical committee blood collection centres (EFS)–INSERM, Paris. Written informed consent for participation was not required for this study in accordance with the national legislation and the institutional requirements (but patients/participants provided written informed consent at the source of EFS). Breeding and maintenance of mice were performed under institutional guidelines, and all of the animal studies and experimental protocols were approved by the animal care and ethics committee of animal experimentation of the IGBMC n°017 and by the Ministère de l’enseignement supérieur, de la recherche et de l’innovation.

## Author contributions

CH and LM conceived and designed mouse study, AK, SB and JB conceived and designed human primary basophil study. CH initiated this study and conducted most experiments and acquired data. PMa contributed to the characterization of Il3-knockout and Il3-conditional knockout mouse lines, and the analyses of IL-3 expression by intracellular staining. PH established FITC model and conducted RA+FITC treatment mouse experiments. AK and SB performed human primary basophil culture and treatment, RNA extraction and cDNA preparation. PMe and EF performed qPCR analyses of human basophils. M-CB contributed to the design and the generation of Il3-knockout and -conditional knockout mouse lines. CH, PMa, PH, AK, SB, PMe, EF, JB and LM analyzed and interpreted data. CH, JB and LM wrote and revised the manuscript. LM directed the study and supervised the work. All authors contributed to the article and approved the submitted version.
