# Corrigendum: IL-3 produced by T cells is crucial for basophil extravasation in hapten- induced allergic contact dermatitis

**DOI:** 10.3389/fimmu.2023.1254157

**Published:** 2023-07-18

**Authors:** Carole El Hachem, Pierre Marschall, Pierre Hener, Anupama Karnam, Srinivasa Reddy Bonam, Pierre Meyer, Eric Flatter, Marie-Christine Birling, Jagadeesh Bayry, Mei Li

**Affiliations:** ^1^ Institut de Génétique et de Biologie Moléculaire et Cellulaire, Centre National de la Recherche Scientifique UMR7104, Institut National de la Santé et de la Recherche Médicale U1258, Université de Strasbourg, Illkirch, France; ^2^ Institut National de la Santé et de la Recherche Médicale, Centre de Recherche des Cordeliers, Sorbonne Université, Université de Paris, Paris, France; ^3^ Institut Clinique de la Souris, Illkirch, France; ^4^ Department of Biological Sciences & Engineering, Indian Institute of Technology Palakkad, Palakkad, India

**Keywords:** basophil, IL-3, allergy, skin, extravasation, integrin, retinoic acid

In the published article, there was an error in the plasmid number and website link.

A correction has been made to **Materials and methods**, *Mice*. This sentence previously stated:

**“**In order to obtain an Il3 “2 in 1” allele (tm1a, Figure S1), we acquired and modified an IMPC plasmid PG00140_W_3_C02 (https://www.mousephenotype.org/data/genes/MGI:1923649)”

The corrected sentence appears below:

“In order to obtain an Il3 “2 in 1” allele (tm1a, Figure S1), we acquired and modified an IMPC plasmid ETPG00275_W_2_F02 (https://www.mousephenotype.org/data/genes/MGI:96552)”

The authors apologize for this error and state that this does not change the scientific conclusions of the article in any way. The original article has been updated.

