# Supplementary material for: IL-3 produced by T cells is crucial for basophil extravasation in hapten-induced allergic contact dermatitis
Source: Front Immunol. 2023 Apr 26;14:1151468. doi: 10.3389/fimmu.2023.1151468 (PMC10169741; doi:10.3389/fimmu.2023.1151468)
Supplement: Supplementary file 1 [file Image_1.pdf]

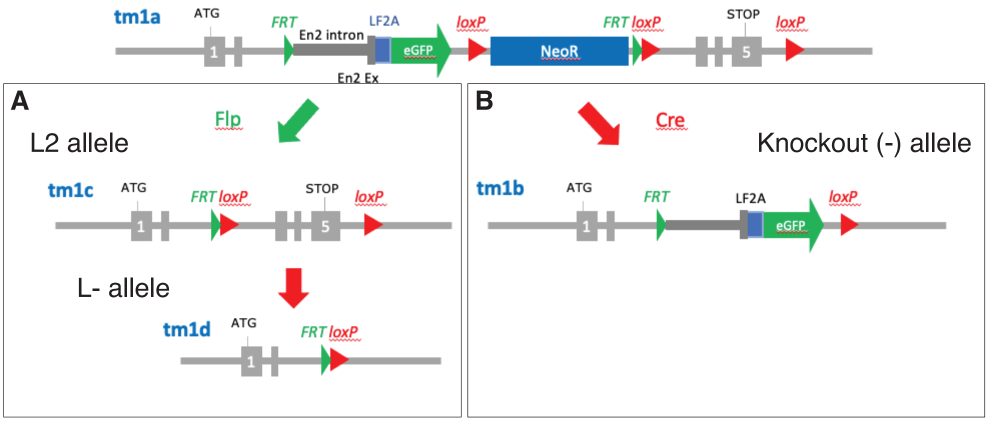

Figure S1. Illustration of *Il3* alleles. The tm1a allele contains the insertion of an eGFP trapping cassette and a floxed promoter-driven neo cassette inserted into the intron 2 of *Il3* gene. Note that this cassette disrupts the Il3 gene function and tag *Il3*. En2, mouse Engrailed 2. Ex, exon. (**A**) Flp converts the tm1a allele to a conditional allele (tm1c; or "L2" allele) with full activity of the gene, which can be converted to tm1c allele (or "L-" allele) when the floxed exons are excised further by Cre. (**B**) Cre deletes the promoter-driven selection cassette and floxed exons of the tm1a allele, leading to a eGFP-tagged allele with the knockout of Exons 3 to 5 (tm1b; or knockout "-" allele). In this paper, $Il3^{tm1c/tm1c}$ is named as $Il3^{L2/L2}$, and $Il3^{tm1b/tm1b}$ is named as $Il3^{-/-}$ mice.

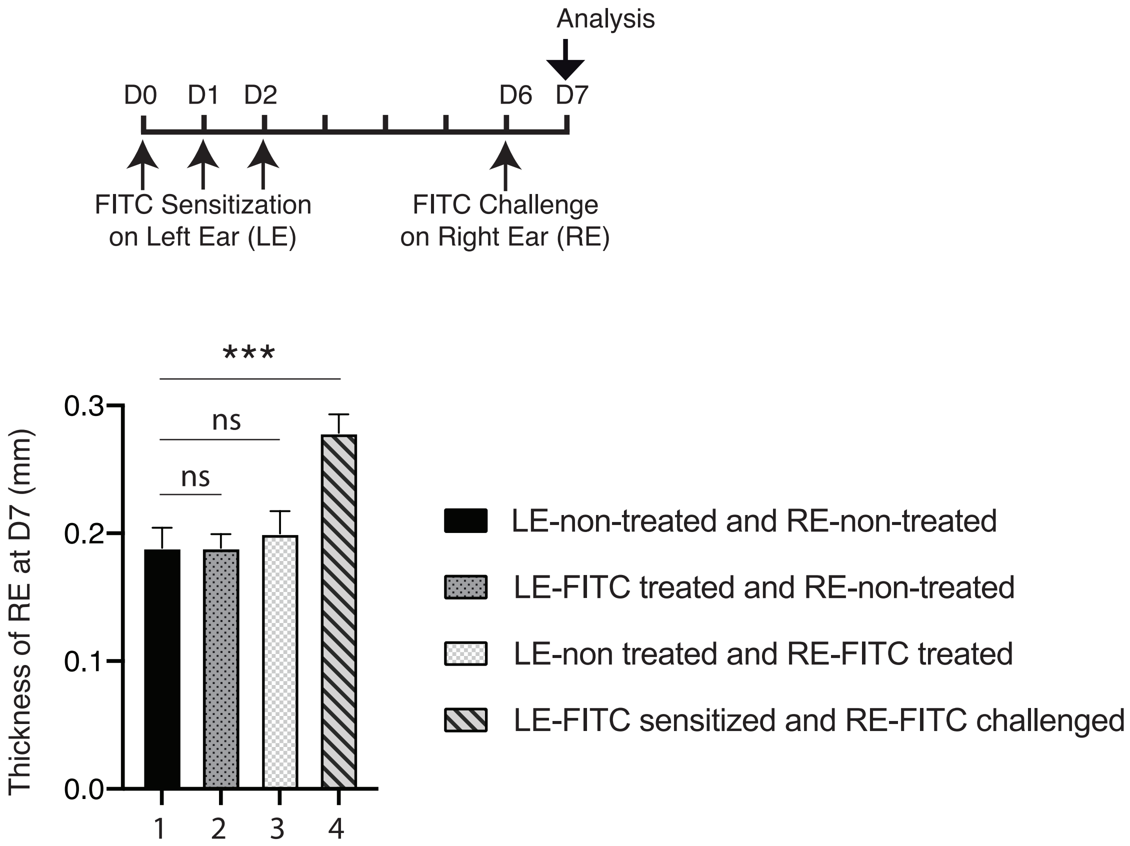

Figure S2. Comparison of the thickness of right ear (RE) at D7. Upper, experimental protocol. Eight to twelve-week-old female mice were treated with FITC on left ear (LE) at Day (D)0, D1 and D2 (sensitization phase). Right ears (RE) were then treated at D6 with FITC (challenge phase) and sampled for analyses at D7. Lower, comparison of thickeness of REs from group-1 (LE-non-treated and RE-non-treated), group-2 (LE-FITC-treated and RE-non-treated), group-3 (LE-non-treated and RE-FITC-treated) and group-4 (LE-FITC sensitized and RE-FITC challenged). ***P≤0.001 (Student's t-test). ns, not significant. Values are mean ± SEM (n=7 mice per group).

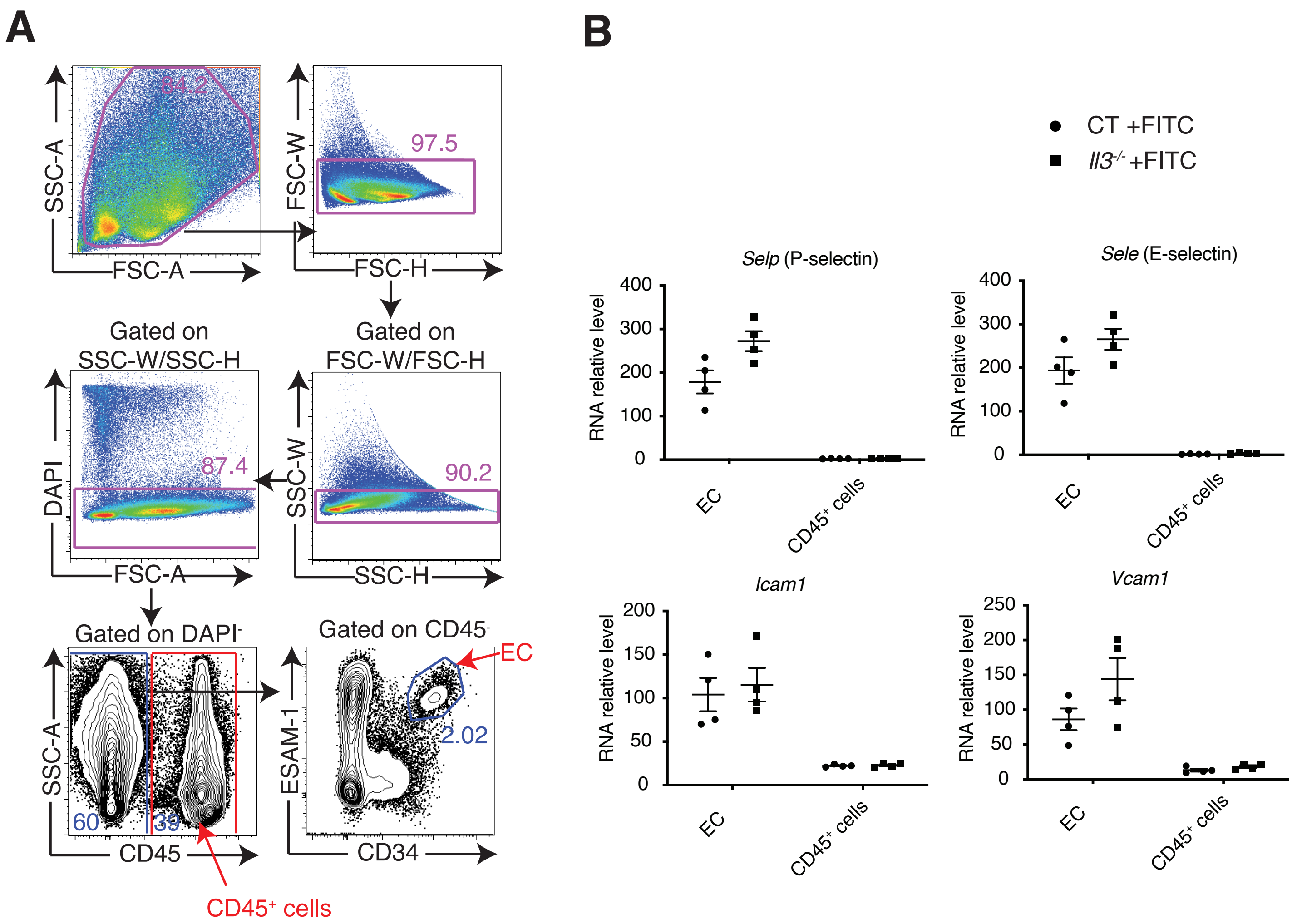

Figure S3. RT-qPCR analyses of FACS-sorted endothelial cells (ECs) and CD45+ hematopoietic cells. (**A**) Gating strategy for sorting of CD45$^-$ESAM-1$^+$CD34$^+$ ECs and CD45$^+$ cells from RE dermis of FITC-treated CT and *Il3*$^{-/-}$ mice. (**B**) RT-qPCR analyses. Values are mean ± SEM (n=4 mice per group).

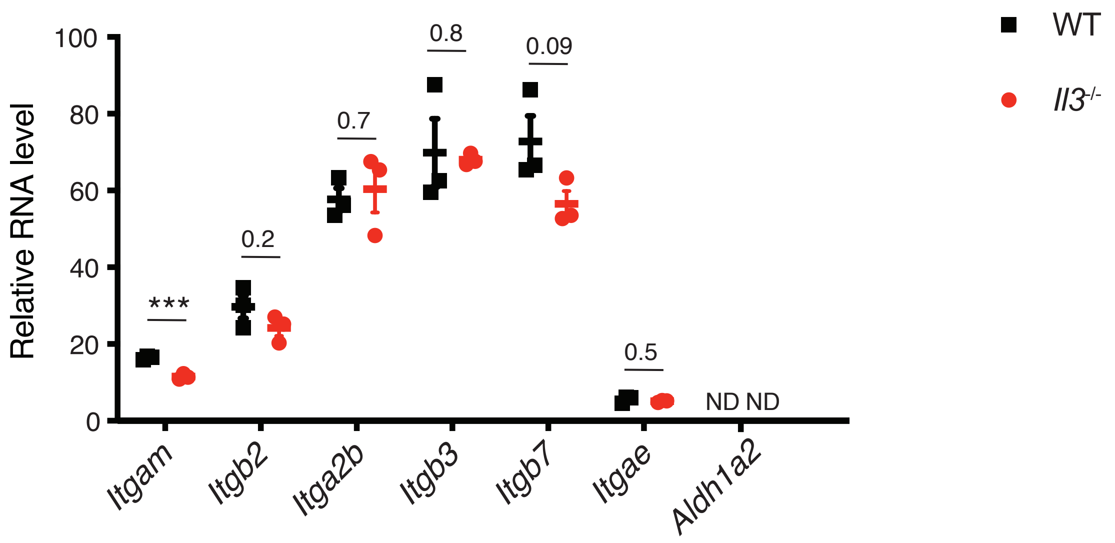

Figure S4. RT-qPCR analyses of naive basophils isolated from wildtype and *Il3*$^{-/-}$ spleens in steady state. Baosphils were FACS sorted from spleens (CD45^{int}CD49b^{+} FcεRIa^{+}) and proceeded for RNA extraction and RT-qPCR analyses, showing that the expression of Itgam, but not Itgb2, Itga2b, Itgb3, Itgb7, Itgae, is significantly lower in *Il3*$^{-/-}$ than wildtype control (CT) mice. ND, non detectable (Cp>50). ***P≤0.001 (Student's t-test). Values are mean ± SEM (n=3 mice per group).
